# The State of Peripheral Blood Natural Killer Cells and Cytotoxicity in Women with Recurrent Pregnancy Loss and Unexplained Infertility 

**DOI:** 10.22074/ijfs.2019.5503

**Published:** 2019-01-06

**Authors:** Azam Azargoon, Yasaman Mirrasouli, Mahdieh Shokrollahi Barough, Mehdi Barati, Parviz Kokhaei

**Affiliations:** 1 Abnormal Uterine Bleeding Research Center, Semnan University of Medical Sciences, Semnan, Iran; 2 Department of Infertility, Amir-AL-Momenin Hospital, Semnan University of Medical Sciences, Semnan, Iran; 3 Student Research Committee, Semnan University of Medical Sciences, Semnan, Iran; 4 Cancer Immunotherapy and Regenerative Medicine Department, Breast Cancer Research Center, Motamed Cancer Institute, ACECR, Tehran, Iran; 5 Department of Immunology, Faculty of Medicine, Mashhad University of Medical Sciences, Mashhad, Iran; 6 Cancer Research Center, Semnan University of Medical Sciences, Semnan, Iran; 7 Immune and Gene therapy Lab, CCK, Karolinska University Hospital Solna, Stockholm, Sweden

**Keywords:** CD56^+^, Infertility, Perforin, Peripheral Natural Killer Cell, Recurrent Miscarriage

## Abstract

**Background:**

The prognostic value of peripheral natural killer (pNK) cells, as a screening test in women with recur- rent pregnancy loss (RPL) and unexplained infertility, is still a matter for discussion. The purpose of this study was to compare the percentage of circulating CD56^+^NK cells, CD69 and perforin markers between women with unexplained infertility and RPL with the healthy control group.

**Materials and Methods:**

In this case-control study, the percentage of CD56^+^NK cells and activation markers (CD69 and perforin levels) in the peripheral blood were measured in 25 women with unexplained infertility, 24 women with idiopathic RPL and 26 women from the healthy control group, using specific monoclonal antibodies by flow cytometry.

**Results:**

The percentage of CD56^+^NK cells was significantly higher in patients with infertility in comparison with the healthy control group (P=0.007). There were not significant differences either in the total number of CD56^+^cells between the RPL group and the control group (P=0.2) or between the RPL group and the infertile group (P=0.36). The percentage of CD69^+^lymphocytes in RPL group was significantly higher than in the infertility group (P=0.004). There was a statistically significant difference in Perforin levels between RLP and control (P=0.001) as well as RPL and infertile (P=0.002) groups.

**Conclusion:**

An increased percentage of CD56^+^NK cells in patients with unexplained infertility, an elevated expression of CD69 on NK cells in patients with RPL and infertility and a high level of perforin on CD56^+^cells in the RPL group might be considered as immunological risk factors in these women.

## Introduction

Infertility is defined as the failure of a couple to get pregnant after 12 months or more of having regular unprotected intercourse. Unexplained infertility is idiopathic and its cause remains unclear when the standard investigation of both male and female partner has made other infertility diagnoses impossible. Recurrent pregnancy loss (RPL), a heterogeneous circumstance often idiopathic, is described as three or more sequential miscarriages occurring before 20 weeks of gestation (1). However, the American Society of Reproductive Medicine (ASRM) has lately defined again RPL as two or more failed pregnancies and the American College of Obstetrician and Gynecologists has stated that the causes of recurrent fetal losses are similar in women who have had two or more miscarriages in comparison with women with three losses (2). The causes of RPL could be chromosomal abnormalities, uterine anomalies, endometrial infections, endocrine etiologies. antiphospholipid syndrome inherited thrombophilias, and alloimmune factors.

Among these suggested causes, only chromosomal abnormalities, antiphospholipid syndrome and uterine anatomic abnormalities are universally approved (3). One of these causes can be seen in about 50% of patients. However, in the remainder the cause is unknown (1, 3).

There is increasing evidence that these cases of unexplained infertility and RPL might have an immunological background. Natural killer (NK) cells which are present in the endometrium at the time of implantation and during early pregnancy seem to play a role in this regard. NK cells are a section of the innate immune system, and constitute 5-10% of peripheral blood lymphocytes (PBL) and 70-90% of uterine lymphocytes. There are two clearly different subgroups of human NK cells identified by cell surface density of CD56 (D56bright or CD56dim). Although both peripheral NK (pNK) and uterine NK (uNK) cells show the surface CD56, pNK cells differ from uNK cells in both phenotype and function and the fact that 10% of pNK cells are similar to uNK cells (4). Moreover, 90% of pNK cells are CD56dim and CD16+, while only 80% of uNK cells are CD56bright and CD16. CD56dim cells have a more toxic activity, although the CD56bright part is the most important source of NK cell-derived immunoregulatory cytokines (5). Obviously uNK cells are important for the success and continuance of pregnancy.

One study reported that pNK cell levels show changes in decidual NK cell levels (6). Whereas some other reports have shown that the assessment of peripheral blood NK cells would not determine the events in the uterus (7, 8). Several studies have tried to find out the relationship between altered pNK cell parameters and RPL. Some case-control studies have discovered a relationship between pNK cell numbers (9, 10) and activity (11-13) with RPL. On the other hand, some studies have shown no difference in pNK cells levels between RPL and controls (14-16). Similarly, there isn’t much information on the relationship between marker of CD69 and RPL. Two different studies reported that NK cells from patients with RPL showed more CD69 than NK cells from controls (9, 17). 

A recent study has not indicated any significant difference in CD96 marker between RPL and controls (13). Also there is not consistency in the association of pNK cells with infertility. Some studies have shown a relationship between pNKcells and infertility (9, 18-20), while some have not (21). A systemic review in 2011 (22) and a large cohort study in 2013 (23) have shown that the prognostic value of analyzing pNK or uNK cell parameters remains doubtful and more researches are needed to accept or deny the role of NK cell measuring as a predictive test for screening women possibly benefiting from immunotherapy. There are few studies which have compared pNKcell numbers and cytotoxicity level at the same time in women with RPL and idiopathic infertility and there are not any studies to measure perforin level in pNK cell in these women. So we decided to determine whether there was a remarkable difference in pNK percentage, CD69 marker and perforin level between women with a history of recurrent miscarriage or unexplained infertility and healthy control women. 

## Materials and Methods

All the samples were taken from patients who came to the clinic of Amir Al-Momenin Hospital, Semnan, Iran from June 2011 to December 2013 for the evaluation of RPL or infertility in a case control study. The Research Council and Ethical Committee of Semnan University of Medical Sciences provided us with the ethical approval and later the informed written consents were collected from patients for this case-controlled study. Seventy five women were included in three age match group in this study (24 with a history of unexplained RPL, 25 with unexplained infertility and 26 healthy women with no history of pregnancy problem, convenient sampling). In the infertility group, women had an infertility history of more than 1 year, normal serum prolactin (PRL) and thyroid function tests (T4 and TSH), documented patent tubes by hysterosalpingography, and had no other infertility factor, and the male partner had a normal sperm count, motility and morphology according to the World Health Organization (WHO 2010) standards. Women with RPL had a history of at least two sequential spontaneous miscarriages. 

Unexplained RPL was defined as a history of =2 sequential miscarriages in which all the following results were normal: parental karyotypes, thyroid function, fetal bovine serum (FBS), anti-cardiolipin antibodies, antiphospholipid antibodies, lupus anticoagulant, follicle-stimulating hormone (FSH), prolactin, progesterone, estrogen, testosterone, free androgen index, prothrombotic risk factors including activated protein-C resistance, factor V Leiden and prothrombin mutations, pelvic ultrasonography and hysterosalpingogram. Twenty six healthy parous women had at least one live birth and had no history of miscarriage, preeclampsia, ectopic pregnancy or preterm delivery.

Sampling: 5 ml of heparinized peripheral blood was taken in mid luteal phase and in women with RPL, at least 2 months after the last abortion. The blood samples were immediately taken to the Immunology Laboratory of Semnan University of Medical Sciences. The whole blood sample was separated into peripheral blood mononuclear cells (PBMC) by ficoll separation and then PBMCs were labeled and kept in freezing condition medium: (RPMI1640+10%FCS+10%DMSO) at the -70°C freezer until all patient samples were collected.

### Flow cytometry analysis

After sampling was completed, the stored cells were thawed and subsequently surface and intracellular staining were performed. Surface markers were determined by flow cytometry, using fluorochrome-conjugated monoclonal antibodies, anti CD3, CD69, CD19, CD56, and perforin using permabilization buffer for permabilizing cell membrane to facilitate antibody entry into cells. Antibodies were bought from BD Biosciences (San Jose, CA, USA) or ebioscience. Appropriate concentrations of antibodies in addition to isotype matched control were added to the cells (5×10^5^ cells/tube) in 100 µL staining buffer and incubated for 25 minutes at 4°C in the dark. Analysis were done by using PARTEC, CyFlow® Space device and FlowMax software. At least 50,000 lymphocyte-gated cells were obtained and analyzed for CD56^+^CD19^+^, perforin+ cells. The criteria for positive staining were set at a fluorescent intensity displayed by <0.5% of the cells stained by the appropriate fluorochrome-conjugated isotype control monoclonal antibodies (mAb). The results and graphs were analyzed using Flowjo version 10A software (Flowjo, USA).

### Statistical analysis

The Kolmogorov-Smirnov test was used to examine the normality of the distributions. A one-way analysis of variance and Tukey’s range test for normally distributed data and Kruskal-Wallis analysis for data with non-normal distribution were used to compare study groups. The results were reported to be statistically significant if the P value was<0.05.

## Results

Mean age of the study population was 29.2 ± 3.4 (mean ± SD) years in infertile group, 28.9 ± 3.2 (mean ± SD) years in RPL group and 28.8 ± 3.3 (mean ± SD) years in control group. There were no significant differences in age distribution among them (P=0.6). 

**Fig.1:**
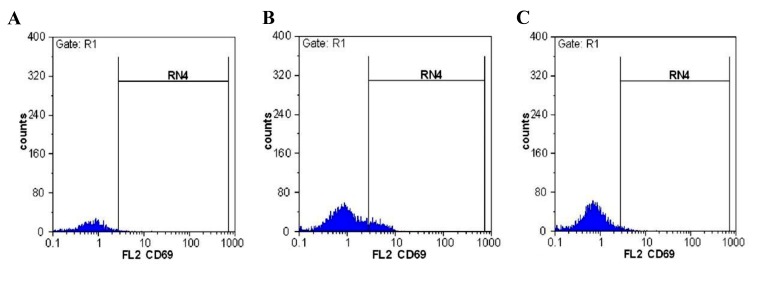
CD69 positive population in CD56^+^ gated cells. A. Control group, B. Recurrent pregnancy loss (RPL) patients, and C. Infertile.

Mean percentage of CD56^+^ cells in infertile, RPL and control groups were respectively: 18.36 ± 7.9, 15.97 ± 5.1, 13.26 ± 5.02. The Mean percentage of peripheral CD56 + cells in the infertile group was remarkably higher (P=0.007) than that of the control subjects. There were not significant differences in the total number of CD56^+^ cells between the RPL group and the control group (P=0.2) and neither between the RPL group and the infertile group (P=0.36, Table 1, Fig .1).

The median percentage of CD69^+^ cells were: 4.5 (1.5-8)% in infertile group, 8 (6-10)% in RPL group, and 6 (4-11)% in control group. The Manne-Whitney analysis between groups showed a significantly higher percentage of CD69^+^ cells in RPL group than the infertile group (P=0.004). However, there were no significant differences between the infertile group (P=0.11) and the RPL group (P=0.1) with the control group. The Perforin positive cells median percentage in the control group was 6.4 (4-8)%, in RPL was 16.5 (9-31)% and in the infertile group was 8 (5-14)%. The Perforin positive cells in infertile group were significantly higher than others (Table 1).

The results showed that 15.8% ± 5.9 of total CD56 cells in patients with RPL and 32% ± 14.4 in the infertile group expressed CD69 as compared with 10.6% ± 5.01 in control group. 

**Table 1:** The percentage of peripheral NK cells (%) and expression of CD69 and perforin levels on these cells in RPL, infertile and controls groups


Cell population	Control	RPL	Infertile	P1	P2	P3

CD56^+^	13.26 ± 5.02	15.97 ± 5.1	18.36 ± 7.9	0.2	0.007	0.36
CD69^+^	6 (4-11)	8 (6-10)	4.5 (1.5-8)	0.1	0.11	0.004
Perforin^+^	6.4 (4-8)	16.5 (9-31)	8 (5-14)	0.001	0.07	0.002
CD56^+^CD69^+^	10.6 ± 5.01	15.8 ± 5.9	32 ± 14.4	0.001	0.001	0.001
CD56^+^Perforin^+^	7 (4-12)	16 (9-23)	8 (5-9)	0.001	0.6	0.001
CD69^+^Perforin^+^	6 (4-10)	10 (6-16)	6.5 (3-9.5)	0.02	0.7	0.01


Values are presented as mean ± SD and median (interquartile range). NK; Natural killer, RPL; Recurrent pregnancy loss, P1; P value: for the difference between mean value in the RPL group and control group, P2; P value: for the difference between mean value in the infertile group and control group, and P3; P value: for the difference between mean value in the infertile group and RPL group.

There was a statistically significant difference in the expression of CD69 in CD56^+^ cells between the control group and RPL group (P=0.001), the infertile group (P=0.001) and between RPL and infertile group (P=0.001, Table 1). This study showed that 16 (9-23)% of total CD56 cells in patients with RPL and 8 (5-9)% in the infertile group expressed perforin as compared with 7 (4-12)% in control group. There was a statistically significant difference in the expression of perforin in CD56^+^ cells between the RPL group compared with the control group (P=0.001) and the infertile group (P=0.001). However, there was not a significant difference in the expression of perforin in CD56^+^ cells the infertile group and the control group (P=0.6). 

The triple staining results showed the CD69^+^Perforin+ population in control group was 6 (4-10)% in RPL group was 10 (6-16)% and in the infertile group was 6.5 (3- 9.5)%. The statistical analysis showed a significant difference between RPL and infertile group and control (P=0.01, P=0.02) without a significant difference between control and Infertile groups (P=0.7).

## Discussion

The findings of this study showed that the levels of CD56^+^ T cells were remarkably higher in infertility group than the control group. But there were no important differences in the total levels of CD56^+^ cells between the RPL group and the other two groups. Moreover, there was a significant increase in the display of CD69 on CD56^+^ cells in the RPL group and the infertile group compared with the control group. We also showed that the level of perforin on CD56^+^ cells significantly increased in the RPL group compared with the other two groups. Findings of this study were similar to those of case-control studies of Emmer et al. (14), Souza et al. (15) and Wang et al. (16). They were also unable to discover a significant difference in pNK parameters between women with RPL and controls. On the other hand, Ntrivalas et al. (9) and Yamada et al. (10) showed a relationship between pNK cell numbers and RPL. Aoki et al. (11) and Shakhar et al. (12) also showed an increased pNK cell activity, using both standard and whole blood assays in women with RPL.

King et al. (13) showed that in women with RPL, the NK percentage was significantly higher and CD56 bright to CD56dim ratio was significantly lower than controls. They also noticed that an NK percentage of 18% was very particular for women with RPL and thus described 12.5% of women with RPL as having high NK percentage, in comparison with 2.9% of controls. Katano et al. (23) in a cohort study on 552 patients with a history recurrent miscarriages showed that high pNK cell activity was found not to be a nondependent risk factor for the next miscarriages. They suggested the clinicians should not consider the NK activity as a systematic RPL investigation, since its clinical importance has not been established yet.

There are also contradictory reports regarding the association of pNK cells with infertility. Some studies have shown a relationship between pNK cells and infertility (18-20). In 1996, Beer et al. (18) showed that women with unexplained infertility and several former in vitro fertilization (IVF) failures showed significantly increased levels of CD56^+^ PBL than normal fertile controls and also reported that the pregnancy rate was much better in those with CD56^+^ levels less than 12%. Matsubayashi et al. (19) also showed a significantly higher NK-cell activity by using a chromium-51 release cytotoxicity assessment in 94 infertile women who, despite the treatment, failed to get pregnant for 6 or more months in comparison with the control group. They continued their study with 77 patients out of 94 who were watched for 2 years, 28 of whom had conceived but 49 had not. They observed that the peripheral NK activity of the group which had got pregnant was significantly lower than that of non-conception group (20). However, Thum et al. (24) and Baczkowski and Kurzawa (25), in two separate studies, compared the percentage of pNK cell in patients with IVF failure with successfully treated IVF cases from the control group. 

They noticed no difference in percentage NK cell and NK cell subpopulation in infertile women who were unable to get pregnant and those who became pregnant after assisted reproductive technology. Tang et al reported a systemic review and came to this conclusion that there was no association between the subsequent pregnancy result and either pNK or uNK cell activity in women with RPL and infertility (22). Recently Seshadri in a meta-analysis showed remarkably higher NK cell numbers or percentages in women with RPL in comparison with the controls. They also noticed that the number of peripheral NK cells was significantly higher in infertile women versus fertile controls. On the other hand, the meta-analysis of studies where uNK cells were measured showed no significant difference in women with RPL versus controls.

They recommended that more research should be conducted before NK cell assessment can be suggested as a diagnostic method in the area of female infertility or RPL. There is no clear reason why the results are different when the information for NK cells is shown as numbers or a percentage. So, they suggested that NK cell measuring and immune therapy should not be recommended except in the area of clinical research (26). CD69 is one of the earliest particular markers of NK cell activity (27, 28). The elevated NK cell CD69 presentation is closely linked with higher cytotoxic activity and target cell lysis (29, 30). In the present study, the expression of CD69 on CD56^+^ cells in the RPL group and infertile group were remarkably higher than the control group. In normal pregnancy, compared to an embryonic pregnancy, NK cell cytotoxicity decreases, suggesting that activated CD69 expressing NK cells have a significant role in controlling trophoblast growth and placental development (31). Ntrivalas et al. (9) showed that women with a history of recurrent miscarriage or unexplained infertility had a significant increase in CD69 expression on CD56 NK cells in comparison with that of normal controls.

In a comparative study of activation and inhibition markers of circulating NK cells, Coulam and Roussev (32) also reported that infertile women had a remarkably more increased expression of NK cell activation markers of CD69C and CD161C than fertile women.

Ghafourian et al. (33) reported that the percentage of NK cells and the expression of CD69, CD94 and CD161 surface markers on CD56^+^NK cells were remarkably more elevated in patients with RPL and in women who had a history of IVF failure than the healthy multiparous and successful IVF control groups. However, Baczkowski and Kurzawa (25) reported there was no difference in CD69 expression on PBL subpopulations including T and B and NK cells among the fertile control group, infertile women who got pregnant and those who did not get pregnant after intracytoplasmic sperm injection. It is a well-known fact that NK cells release both perforin and serine proteases such as granzyme B upon target cell contact (34, 35). It has been hypothesized that granzyme B induces apoptosis of the target cell in the presence of perforin (36, 37). In this study we showed that the level of perforin on CD56^+^ cells significantly increased in the RPL group compared with the other two groups. Yamada et al. (38) showed a small increase in perforin-positive uNK cells in human spontaneous miscarriage with a normal fetal chromosomal karyotype. On the other hand, Nakashima showed that the number of granulysin-positive CD56bright uNK cells was remarkably higher in the decidua basalis in spontaneous miscarriage than in normal pregnancy, although he did not notice any difference in the numbers of perforin- positive and granzyme B-positive cells (39).

## Conclusion

The findings of this study showed a significant increase in the percentage of CD56^+^ pNK cells among the infertility group and also a significantly higher level of CD69 expression on CD56^+^ NK cells in women with RPL and unexplained infertility in comparison with healthy control group. We also showed that the level of perforin on CD56^+^ cells significantly increased in the RPL group compared with the other two groups. Although it can be considered as immunological risk markers in these women, the prognostic value of PNK number assessment or activity remains still doubtful. So because of many arguments in this field, further researches are needed to accept or deny the role of NK cell evaluation as a predictive test for screening women with infertility or RPL. 

## References

[1] Practice Committee of American Society for Reproductive Medicine (2013). Definitions of infertility and recurrent pregnancy loss: a committee opinion. Fertil Steril.

[2] American College of Obstetricians and Gynecologists (2002). ACOG practice bulletin.Management of recurrent pregnancy loss.Number 24, February 2001.(Replaces Technical Bulletin Number 212, September 1995).American College of Obstetricians and Gynecologists. Int J Gynaecol Obstet.

[3] Lee RM, Silver RM (2000). Recurrent pregnancy loss: summary and clinical recommendations. Semin Reprod Med.

[4] Moffett-King A (2002). Natural killer cells and pregnancy. Nat Rev Immunol.

[5] Deniz G, Christmas SE, Brew R, Johnson PM (1994). Phenotypic and functional cellular differences between human CD3- decidual and peripheral blood leukocytes. J Immunol.

[6] Park DW, Lee HJ, Park CW, Hong SR, Kwak-Kim J, Yang KM (2010). Peripheral blood NK cells reflect changes in decidual NK cells in women with recurrent miscarriages. Am J Reprod Immunol.

[7] Saito S (2000). Cytokine network at the feto-maternal interface. J Reprod Immunol.

[8] Moffett A, Regan L, Braude P (2004). Natural killer cells, miscarriage, and infertility. BMJ.

[9] Ntrivalas EI, Kwak-Kim JY, Gilman-Sachs A, Chung-Bang H, Ng SC, Beaman KD (2001). Status of peripheral blood natural killer cells in women with recurrent spontaneous abortions and infertility of unknown aetiology. Hum Reprod.

[10] Yamada H, Morikawa M, Kato EH, Shimada S, Kobashi G, Minakami H (2003). Pre-conceptional natural killer cell activity and percentage as predictors of biochemical pregnancy and spontaneous abortion with normal chromosome karyotype. Am J Reprod Immunol.

[11] Aoki K, Kajiura S, Matsumoto Y, Ogasawara M, Okada S, Yagami Y (1995). Preconceptional natural-killer-cell activity as a predictor of miscarriage. Lancet.

[12] Shakhar K, Ben-Eliyahu S, Loewenthal R, Rosenne E, Carp H (2003). Differences in number and activity of peripheral natural killer cells in primary versus secondary recurrent miscarriage. Fertil Steril.

[13] King K, Smith S, Chapman M, Sacks G (2010). Detailed analysis of peripheral blood natural killer (NK) cells in women with recurrent miscarriage. Hum Reprod.

[14] Emmer PM, Nelen WL, Steegers EA, Hendriks JC, Veerhoek M, Joosten I (2000). Peripheral natural killer cytotoxicity and CD56(pos)CD16(pos) cells increase during early pregnancy in women with a history of recurrent spontaneous abortion. Hum Reprod.

[15] Souza SS, Ferriani RA, Santos CM, Voltarelli JC (2002). Immunological evaluation of patients with recurrent abortion. J Reprod Immunol.

[16] Wang Q, Li TC, Wu YP, Cocksedge KA, Fu YS, Kong QY (2008). Reappraisal of peripheral NK cells in women with recurrent miscarriage. Reprod Biomed Online.

[17] Prado-Drayer A, Teppa J, Sanchez P, Camejo MI (2008). Immunophenotype of peripheral T lymphocytes, NK cells and expression of CD69 activation marker in patients with recurrent spontaneous abortions, during the mid-luteal phase. Am J Reprod Immunol.

[18] Beer AE, Kwak JY, Ruiz JE (1996). Immunophenotypic profiles of peripheral blood lymphocytes in women with recurrent pregnancy losses and in infertile women with multiple failed in vitro fertilization cycles. Am J Reprod Immunol.

[19] Matsubayashi H, Hosaka T, Sugiyama Y, Suzuki T, Arai T, Kondo A (2001). Increased natural killer-cell activity is associated with infertile women. Am J Reprod Immunol.

[20] Matsubayashi H, Shida M, Kondo A, Suzuki T, Sugi T, Izumi S (2005). Preconception peripheral natural killer cell activity as a predictor of pregnancy outcome in patients with unexplained infertility. Am J Reprod Immunol.

[21] Vujisić S, Lepej SZ, Aksamija A, Jerković L, Sokolić B, Kupesić S (2004). B- and T-cells in the follicular fluid and peripheral blood of patients undergoing IVF/ET procedures. Am J Reprod Immunol.

[22] Tang AW, Alfirevic Z, Quenby S (2011). Natural killer cells and pregnancy outcomes in women with recurrent miscarriage and infertility: a systematic review. Hum Reprod.

[23] Katano K, Suzuki S, Ozaki Y, Suzumori N, Kitaori T, Sugiura-Ogasawara M (2013). Peripheral natural killer cell activity as a predictor of recurrent pregnancy loss: a large cohort study. Fertil Steril.

[24] Thum MY, Bhaskaran S, Bansal AS, Shehata H, Ford B, Sumar N (2005). Simple enumerations of peripheral blood natural killer (CD56+ NK) cells, B cells and T cells have no predictive value in IVF treatment outcome. Hum Reprod.

[25] Baczkowski T, Kurzawa R (2007). Immunophenotypic profiles of peripheral blood lymphocytes on the day of embryo transfer in women undergoing in vitro fertilization. Folia Histochem Cytobiol.

[26] Seshadri S, Sunkara SK (2014). Natural killer cells in female infertility and recurrent miscarriage: a systematic review and meta-analysis. Hum Reprod Update.

[27] Llera AS, Viedma F, Sanchez-Madrid F, Tormo J (2001). Crystal structure of the C-type lectin-like domain from the human hematopoietic cell receptor CD69. J Biol Chem.

[28] Marzio R, Mauël J, Betz-Corradin S (1999). CD69 and regulation of the immune function. Immunopharmacol Immunotoxicol.

[29] De Maria R, Cifone MG, Trotta R, Rippo MR, Festuccia C, Santoni A (1994). Triggering of human monocyte activation through CD69, a member of the natural killer cell gene complex family of signal transducing receptors. J Exp Med.

[30] Lanier LL, Buck DW, Rhodes L, Ding A, Evans E, Barney C (1988). Interleukin 2 activation of natural killer cells rapidly induces the expression and phosphorylation of the Leu-23 activation antigen. J Exp Med.

[31] Ho HN, Chao KH, Chen CK, Yang YS, Huang SC (1996). Activation status of T and NK cells in the endometrium throughout menstrual cycle and normal and abnormal early pregnancy. Hum Immunol.

[32] Coulam CB, Roussev RG (2003). Correlation of NK cell activation and inhibition markers with NK cytoxicity among women experiencing immunologic implantation failure after in vitro fertilization and embryo transfer. J Assist Reprod Genet.

[33] Ghafourian M, Karami N, Khodadadi A, Nikbakht R (2014). Increase of CD69, CD161 and CD94 on NK cells in women with recurrent spontaneous abortion and in vitro fertilization failure. Iran J Immunol.

[34] Young JD, Hengartner H, Podack ER, Cohn ZA (1986). Purification and characterization of a cytolytic pore-forming protein from granules of cloned lymphocytes with natural killer activity. Cell.

[35] Sheikhi AK, Tayade C, Paffaro VA, Croy BA (2007). Are natural killer cells distributed in relationship to nerve fibers in the pregnant mouse uterus?. Pak J Biol Sci.

[36] Trapani JA, Browne KA, Smyth MJ, Jans DA (1996). Localization of granzyme B in the nucleus.A putative role in the mechanism of cytotoxic lymphocyte-mediated apoptosis. J Biol Chem.

[37] Shi L, Mai S, Israels S, Browne K, Trapani JA, Greenberg AH (1997). Granzyme B (GraB) autonomously crosses the cell membrane and perforin initiates apoptosis and GraB nuclear localization. J Exp Med.

[38] Yamada H, Shimada S, Morikawa M, Iwabuchi K, Kishi R, Onoe K (2005). Divergence of natural killer cell receptor and related molecule in the decidua from sporadic miscarriage with normal chromosome karyotype. Mol Hum Reprod.

[39] Nakashima A, Shiozaki A, Myojo S, Ito M, Tatematsu M, Sakai M (2008). Granulysin produced by uterine natural killer cells induces apoptosis of extravillous trophoblasts in spontaneous abortion. Am J Pathol.

